# Seasonal Effects on Bell’s Palsy: Four-Year Study and Review of the Literature

**Published:** 2012-08-30

**Authors:** Hüseyin Narcı, Bahriye Horasanlı, Murat Uğur

**Affiliations:** 1 Department of Emergency, Baskent University Faculty of Medicine, Konya, Turkey; 2 Department of Neurology, Baskent University Faculty of Medicine, Konya, Turkey

Dear Editor,

Bell’s palsy is the sudden onset of unilateral dysfunction of the seventh cranial nerve that results in the paralysis of the facial muscles on the affected side of the [1]. Seasonal distribution have been discussed in several studies with variable, often contradictory, results [2][3]. The aim of this study was to determine whether the Bell’s palsy visits to our hospital exhibit seasonal patterns.

In this retrospective study, we reviewed all cases documented in the archive of our hospital during the period from january 1, 2007, until december 31, 2010.Patient distribution patterns by season, month and age groups were recorded. Data were entered onto SPSS 15.0 for statistical analysis. The Kolmogorov-Smirnov test was used in data analysis, with significance set at P < 0.01.

**Figure 1:**
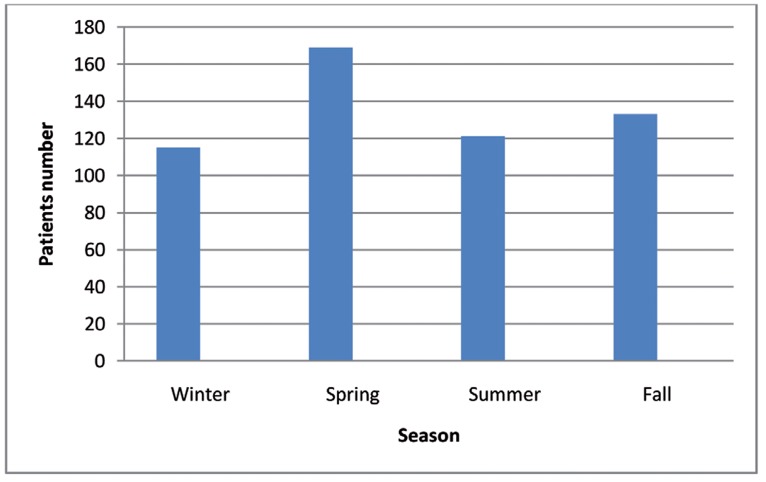
Seasonal distrubition of BP patients

A Total 634 facial paralysis patients visits were established during the study period and 533(84%) of them were diagnosed as BP. The mean age of the BP patients was 55 ± 24.7 years . 51.40% of all patients were males and 48.59% were females. The cases determined as BP, 105 (19.69%) were aged 30-39 years, 83 (15.57%) cases were aged 20-29 years, 50 (9.38%) 70-79 years, 12 (2.25%) 80-89 years and 2 (0.37%) 0-9 years. BP was most common in the 30-39 age group and this was also statistically significant (P < 0.01) (range 9-89). The months with the greatest BP were May (n = 59, 11.06%), March (n = 54, 10.13%), April (n = 51, 9.56%) September (n = 50, 9.38%). July (n = 34, 6.38%), December (n = 34, 6.38%) January (n = 35, 6.56%) and November (n = 36, 6.75%) had the lowest BP (P > 0.01) (range 34-59). Numbers of admitted patients with BP at seasons were winter (n = 115, 21.57%), Spring (n = 169, 31.70%), summer (n = 121, 22.70%) and fall (n = 133, 24.95%) (Figure 1). The peak ratio of BP according to season was in spring and nadir was in winter (t = 11.122, df = 3, P = 0.002) (range 115-169).

The worldwide incidence of BP varies between 11.5 and 40.2 cases per 100,000 person. The greatest incidence has been observed in Japanese, İsraelis and the mexican population, while the smallest in the swedish people [4]. In our study the annual incidence of BP per 100,000 population was found to be 12,7. In previous studies of the incidence of BP, the largest group of patients was found between 15 and 45 years of age [2]. In our study we observed, the age peak incidence occurs between 15 and 45 years.

In a study of 500 patients carried out by Park et al. It is reported that found no significant seasonal distribution of BP cases [5]. Adour et al. did not observe significant differences in the cases of BP occurring during the cold and warm seasons [6].In a study carried out by Peitersen it is reported that found no significant differences from month to month and thus no seasonal variation [7].Spengos et al. reported that observed a decline during the summer, in contrast to peak during the autumn and winter in the cases of BP [4].In our study, the increase in the incidence of the BP in spring is statisticaly significantly. The incidence of the upper respiratory tract infections is increased in spring. The relationship of the BP with the seasons may be related to the increaesed HSV-1 virus activations.We found significant statistical relation between seasonal variation and BP. The risk of BP is high during the spring and low during the winter and summer.
